# Transcriptome data of temporal and cingulate cortex in the Rett syndrome brain

**DOI:** 10.1038/s41597-020-0527-2

**Published:** 2020-06-19

**Authors:** Kimberly A. Aldinger, Andrew E. Timms, James W. MacDonald, Hanna K. McNamara, Jennifer S. Herstein, Theo K. Bammler, Oleg V. Evgrafov, James A. Knowles, Pat Levitt

**Affiliations:** 1 Zilkha Neurogenetic Institute, Keck School of Medicine, University of Southern California, Los Angeles, CA USA; 2 Center for Integrative Brain Research, Seattle Children’s Research Institute, Seattle, WA USA; 3 Center for Developmental Biology and Regenerative Medicine, Seattle Children’s Research Institute, Seattle, WA USA; 4 Department of Environmental and Occupational Health Sciences, School of Public Health, University of Washington, Seattle, WA USA; 5 Department of Psychiatry and the Behavioral Sciences, Keck School of Medicine, University of Southern California, Los Angeles, CA USA; 6 Present Address: Department of Cell Biology, SUNY Downstate Medical Center, Brooklyn, NY USA; 7 Department of Pediatrics and Program in Developmental Neuroscience and Developmental Neurogenetics, The Saban Research Institute, Children’s Hospital Los Angeles, Keck School of Medicine, University of Southern California, Los Angeles, CA USA

**Keywords:** Genetics research, Brain, Paediatric neurological disorders, Autism spectrum disorders, Gene expression

## Abstract

Rett syndrome is an X-linked neurodevelopmental disorder caused by mutation in the methyl-CpG-binding protein 2 gene (MECP2) in the majority of cases. We describe an RNA sequencing dataset of postmortem brain tissue samples from four females clinically diagnosed with Rett syndrome and four age-matched female donors. The dataset contains 16 transcriptomes, including two brain regions, temporal and cingulate cortex, for each individual. We compared our dataset with published transcriptomic analyses of postmortem brain tissue from Rett syndrome and found consistent gene expression alterations among regions of the cerebral cortex. Our data provide a valuable resource to explore the biology of the human brain in Rett syndrome.

## Abstract (summary)

**Table:** 

Measurement(s)	transcriptome • RNA
Technology Type(s)	RNA sequencing
Factor Type(s)	Rett syndrome brain versus control brain • brain regions (temporal cortex and cingulate cortex)
Sample Characteristic - Organism	Homo sapiens

Machine-accessible metadata file describing the reported data: 10.6084/m9.figshare.12383291

## Background & Summary

Rett syndrome (RTT) is an X-linked neurodevelopmental disorder mostly caused by heterozygous *de novo* mutation in the methyl-CpG-binding protein 2 gene (*MECP2*) and predominantly affecting females^1^. *MECP2* duplications have been identified in males with developmental encephalopathy, seizures, autistic features, and recurrent infection^2^. These clinical disorders illustrate the critical requirement for proper *MECP2* expression in human brain development, though how MeCP2 dysfunction leads to the RTT phenotype is unclear.

MeCP2 acts as a global transcriptional regulator by recruiting chromatin-remodeling complexes or regulating higher-order chromatin structures^3–8^. Thus, MeCP2 may be required for fine-tuning the gene expression for a network of protein-coding genes through both direct and indirect mechanisms. Consistent with this hypothesis, small magnitude changes in gene expression have been detected in brain tissue from either human postmortem RTT samples or mouse *Mecp2*-mutants^9–12^. However, most transcriptional studies of postmortem RTT brain have used microarray platforms with small numbers and a lack of age-matched control samples, which impact the sensitivity for detecting transcriptional changes. One study used both microarrays and RNA sequencing (RNA-seq) to examine frontal and temporal cortex from individuals with RTT compared to controls and identified over 200 differentially expressed genes after normalizing data for neuron versus glia composition of samples^13^. Another larger study used RNA-seq to examine motor cortex and cerebellum and identified over 2,000 differentially expressed genes with a global increase in expression^14^.

We generated RNA-seq data using brain samples for two distinct brain regions, temporal cortex and cingulate cortex, from four female RTT and four age-matched female donors. Reduced volume and dendritic branching of neurons in the temporal cortex and reduced connectivity of the cingulate cortex have been reported in RTT, indicating the importance of these brain regions in the disorder^15–18^. We also compared our data with the transcriptomic profiles of RTT brain samples from published RNA-seq datasets^13,14^. The composite analysis will be useful to facilitate interpretation and further understanding of MECP2-mediated changes in human brain.

## Methods

### Brain samples

**Fig. 1:**
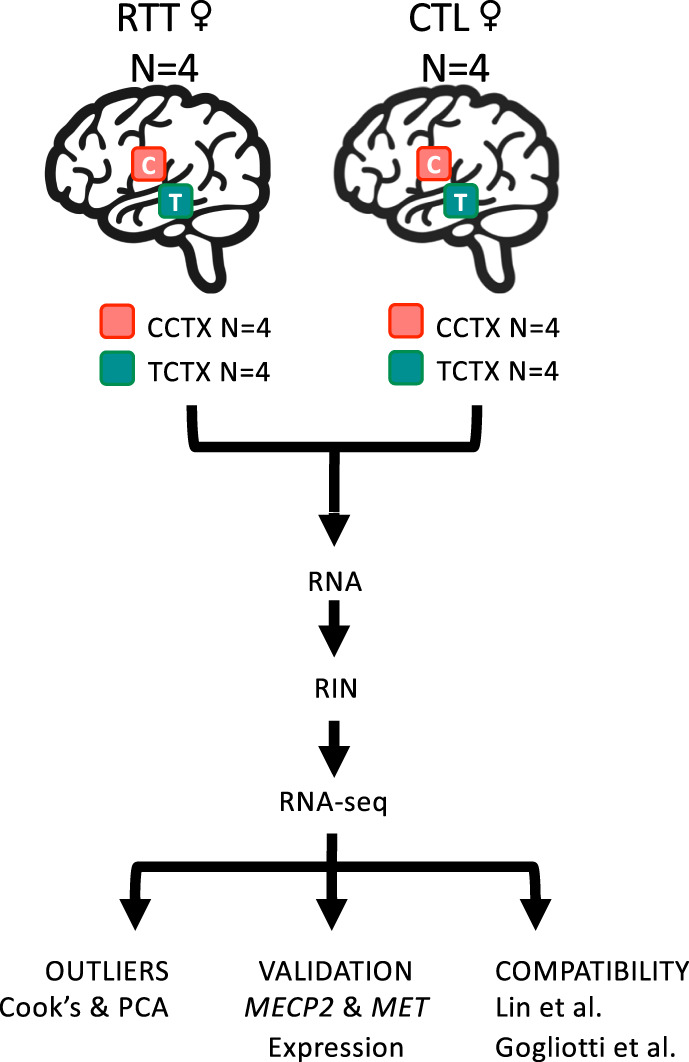
Overview of the experimental workflow.

Postmortem brain tissue samples were obtained from the Harvard Brain Bank (http://hbtrc.mclean.harvard.edu/) and the National Institutes of Health (NIH) NeuroBioBank (https://neurobiobank.nih.gov), with approval from the coordinating foundation (https://www.rettsyndrome.org). Consent was obtained from next of kin and tissue was collected with approval from the Partners Human Research Committee for the Harvard Brain Bank and from The University of Maryland Institutional Review Board (IRB) and The Maryland Department of Health and Mental Hygiene IRB for the NeuroBioBank. Work was approved by the University of Southern California and is compliant with all ethical regulations. Frozen temporal (BA36/38) and cingulate cortex samples were obtained from four RTT and four control (CTL) brain donors that were matched in age (Fig. 1). The Harvard Brain Bank sequenced *MECP2* coding exons and reported intragenic mutations in two of the four brains. Brain donor characteristics are described in Table 1.

**Table 1:** Brain Donor Characteristics.

Brain	Group	Gender	Age	PMI	Source	*MECP2*	
						NM_004992.3	
						cDNA	Protein
1038	CTL	F	24	7	NBB	NA	NA
1614	CTL	F	27	18	NBB	NA	NA
4724	CTL	F	16	15	NBB	NA	NA
4725	CTL	F	32	17	NBB	NA	NA
6355	RTT	F	16	28	HBTRC	NR	NR
7773	RTT	F	24	25	HBTRC	c.473 C > T	p.Thr158Met
7783	RTT	F	26	39	HBTRC	Exon Del	
B7992	RTT	F	31	30	HBTRC	NR	NR

Abbreviations: CTL, control; F, female; HBTRC, Harvard Brain Tissue Resource Center; NA, not applicable; NBB, NIH NeuroBioBank; NR, no mutation in *MECP2* reported; PMI, postmortem interval in hours; RTT, Rett syndrome.

### *MECP2* variant confirmation

Genomic DNA was isolated from brain samples for 7773 and 7783 using the PureLink Genomic DNA Kit (LifeTechnologies) according to the manufacturer’s protocol. We performed Sanger sequencing of *MECP2* to verify the reported variants (Table 1). Chromatograms were aligned to *MECP2* (ENSG00000169057) using MAFFT v7^19^. No additional genes were screened.

### RNA sample and library preparation

Total RNA was previously isolated using the Qiagen RNeasy Kit according to the manufacturer’s instructions^20^. Double stranded cDNA fragments were synthesized from mRNA, ligated with adapters, and size-selected for library construction according to the TruSeq Sample Preparation v2 protocol using 0.5–1.5 μg of total RNA (Table 2). ERCC RNA spike-in controls were not included in this experiment. Library quality was measured using an Agilent 2100 Bioanalyzer and concentration was assessed by PicoGreen incorporation. Barcoded libraries were pooled and sequenced in two lanes using an Illumina HiSeq 2000 sequencer.

**Table 2:** RNA Sample Characteristics.

Brain	Group	Region	RIN	RNA (μg)	GEO	SRA
1038	CTL	CCTX	9.8	1.50	GSM3673208	SRX5527579
1614	CTL	CCTX	9.2	0.70	GSM3673209	SRX5527580
4724	CTL	CCTX	9.1	0.60	GSM3673210	SRX5527581
4725	CTL	CCTX	9.5	1.50	GSM3673211	SRX5527582
6355	RTT	CCTX	8.6	0.60	GSM3673212	SRX5527583
7773	RTT	CCTX	9.8	1.50	GSM3673213	SRX5527584
7783	RTT	CCTX	8.6	0.70	GSM3673214	SRX5527585
B7992	RTT	CCTX	10	1.50	GSM3673215	SRX5527586
1038	CTL	TCTX (BA38)	10	1.50	GSM3673216	SRX5527587
1614	CTL	TCTX (BA36)	8.3	0.51	GSM3673217	SRX5527588
4724	CTL	TCTX (BA38)	9.7	1.28	GSM3673218	SRX5527589
4725	CTL	TCTX (BA38)	9.3	1.00	GSM3673219	SRX5527590
6355	RTT	TCTX	8.6	1.28	GSM3673220	SRX5527591
7773	RTT	TCTX	9.7	1.50	GSM3673221	SRX5527592
7783	RTT	TCTX	8.7	0.51	GSM3673222	SRX5527593
B7992	RTT	TCTX	9.6	1.00	GSM3673223	SRX5527594

Abbreviations: BA, Brodmann area; CCTX, cingulate cortex; CTL, control; RIN, RNA integrity number; RNA, quantity of total RNA used as input for library preparation; RTT, Rett syndrome; TCTX, temporal cortex.

### RNA-Seq data analysis

Single-end reads (100 bp) were aligned to the *Human* reference genome (NCBI build 37/hg19) using STAR v2.5.3a^21^ (see Code Availability 1). Aligned reads mapping to the exons of a gene were summarized into gene counts using featureCounts v1.6^22^ (see Code Availability 2). Picard CollecteRnaSeqMetrics was used to measure the 3′ bias of genes in the RNA-seq data (see Code Availability 3). Gene-level differential expression was analyzed using DESeq2^23^ specifying ~ region + group + bias as the experimental design (see Code Availability 4). Aligned reads mapping to *MECP2* isoforms were also summarized using featureCounts v1.6^22^ (see Code availability 2) by substituting isoforms for gene name.

## Data Records

Count matrix and normalized count matrix were submitted to the NCBI Gene Expression Omnibus (GEO) under accession number GSE128380^24^. The raw FASTQ files can be downloaded from the Sequence Read Archive (SRA) under accession number SRP188555^25^.

## Technical Validation

### *MECP2* variant confirmation

We verified the presence of the *MECP2* c.473 C > T (p.Thr158Met) intragenic variant using DNA isolated from brain 7773 (Supplemental Fig. 1). No *MECP2* variants were detected in exons 2–4 of brain 7783. Since we were unable to amplify exon 1 in 7783, we infer exon 1 is likely to be the deleted exon. We also examined RNA-seq data for presence of *MECP2* variants (Supplemental Fig. 2). The *MECP2* c.473 C > T (p.Thr158Met) intragenic variant was also detected in RNA-seq data from CCTX and TCTX for brain 7773. *MECP2* variants were not detected in RNA-seq data for other RTT brain samples, possibly due to low sequencing read depth of *MECP2* (Supplemental Fig. 3), or because causal variants are present in another gene^26,27^.

### RNA and data quality

**Fig. 2:**
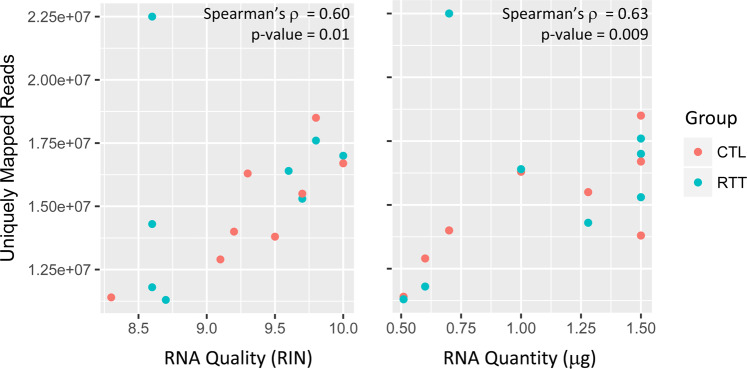
RNA quality or RNA quantity versus number of uniquely mapped reads.

**Fig. 3:**
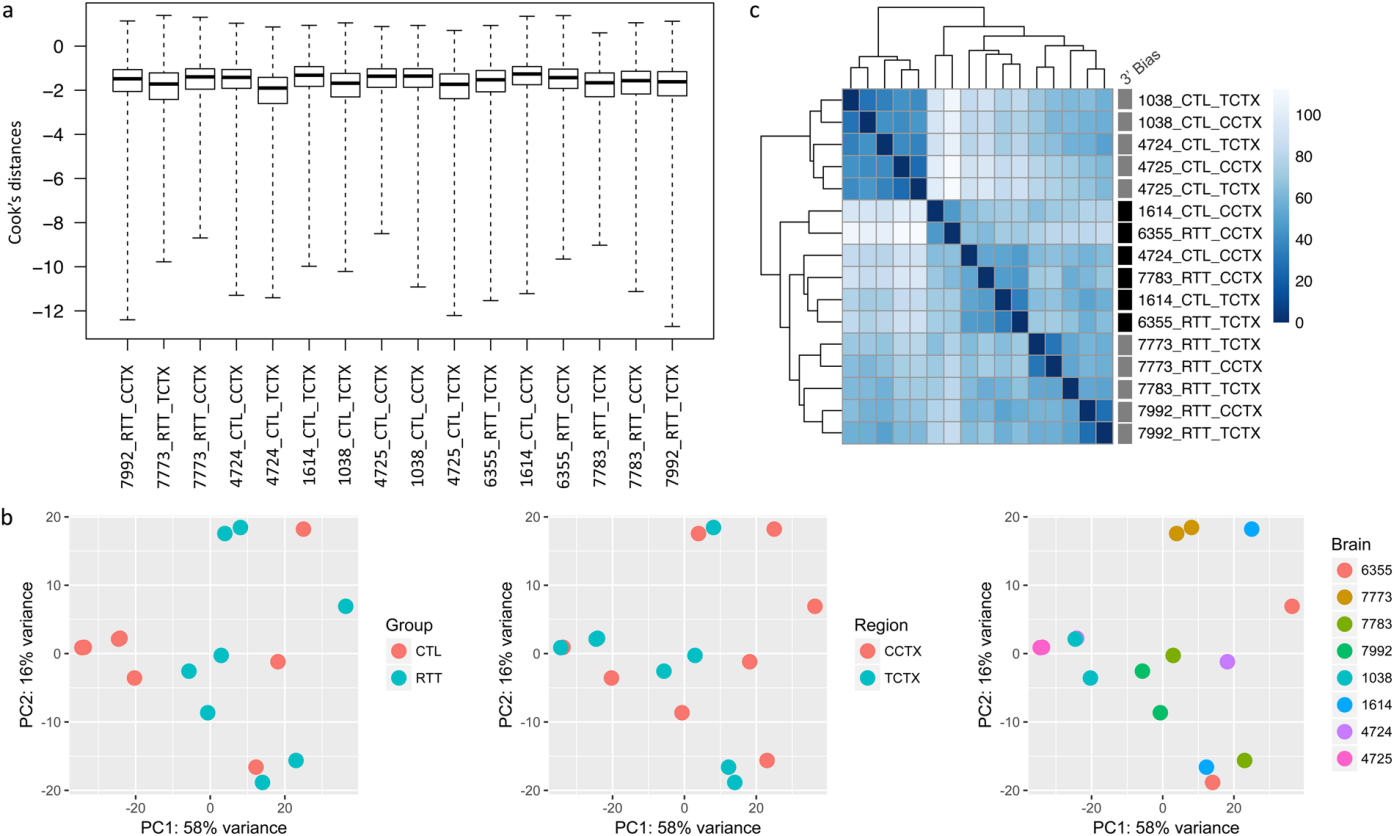
RNA-seq data quality assessment. (**a**) Boxplots showing Cook’s distance calculated for each sample. (**b**) Principal component analysis with samples colored by diagnostic group (CTL, RTT), brain region (CCTX, TCTX), or brain donor. (**c**) Heatmap of the sample distance matrix. Presence (black) or absence (grey) of 3′ bias in RNA-seq data is indicated for each sample.

RNA quality was determined using the Agilent 2100 Bioanalyzer and the RNA 6000 Pico Kit and high-quality RNA was obtained from all samples (RNA integrity number [RIN] > 8.0; median RIN = 9.4 [Table 2]). At the time the experiment was performed, the TruSeq RNA Sample Prep v2 protocol (Part # 15026495 Rev.C, May 2012) was optimized for 0.1–4 μg of total RNA. Although the quantity of RNA input varied among the samples in our experiment, it was equivalent within each age- and tissue- matched case-control sample pair, and all samples were within the optimized range. On average, RNA-seq generated 21.9 million high-quality reads per sample, 70.3% of which mapped uniquely to the *Human* reference genome (NCBI build 37/hg19) (Table 3). RIN and RNA quantity were each correlated with the number of uniquely mapped reads (Fig. 2). Cook’s distance was calculated to test for outliers, with none detected (Fig. 3a). The first principal component explained over 50% of the variance (Fig. 3b). A correlation matrix based on the gene expression data indicated that samples mostly cluster by individual and diagnostic group, but also by 3′ bias (Fig. 3c).

**Table 3:** RNA-seq Data Mapping Statistics.

Brain	Group	Region	Total Reads	% Bases ≥ Q30	Mean Base Quality	Uniquely Mapped Reads	Mapping Rate	Median 5′ to 3′ Bias
1038	CTL	CCTX	22,666,185	77.09	31.71	18,508,346	81.66	2.16
1614	CTL	CCTX	20,406,028	76.18	31.42	13,966,751	68.44	0.76
4724	CTL	CCTX	17,775,778	76.21	31.45	12,903,748	72.59	0.51
4725	CTL	CCTX	17,152,400	76.58	31.57	13,787,313	80.38	1.36
6355	RTT	CCTX	16,799,774	76.38	31.49	11,754,027	69.96	0.71
7773	RTT	CCTX	22,119,416	77.01	31.70	17,627,391	79.69	1.36
7783	RTT	CCTX	32,207,239	77.16	31.72	22,511,241	69.89	0.43
B7992	RTT	CCTX	21,882,359	76.58	31.56	17,048,617	77.91	1.09
1038	CTL	TCTX (BA38)	23,689,678	77.72	31.88	16,671,042	70.37	1.34
1614	CTL	TCTX (BA36)	17,569,738	78.01	31.97	11,367,085	64.70	0.44
4724	CTL	TCTX (BA38)	22,429,527	77.84	31.93	15,519,596	69.19	0.98
4725	CTL	TCTX (BA38)	24,262,545	78.03	31.97	16,322,635	67.28	1.04
6355	RTT	TCTX	25,330,544	76.95	31.64	14,294,799	56.43	0.36
7773	RTT	TCTX	22,557,491	77.85	31.92	15,330,591	67.96	0.97
7783	RTT	TCTX	17,060,381	77.82	31.92	11,301,790	66.25	0.82
B7992	RTT	TCTX	26,211,057	77.11	31.70	16,434,103	62.70	1.01

### *MECP2* and *MET* differential expression

**Fig. 4:**
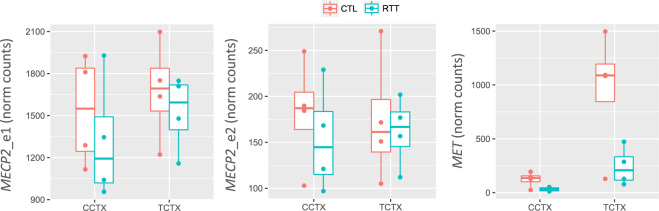
Boxplots showing the expression of *MECP2_e1*, *MECP2_e2*, and *MET* in RTT and CTL brain. Expression values are shown as normalized counts.

We previously used quantitative reverse transcription PCR to compare expression of *MECP2_e1* (NM_004992.3)*, MECP2_e2* (NM_001110792.1), and *MET* (NM_000245.3) in the temporal cortex between RTT and CTL brains^28^. Consistent with our previous results, the RNA-seq data showed no significant difference in *MECP2* expression between RTT and CTL brains (FDR adjusted p-value = 0.16 and 0.59, respectively), while *MET* expression was significantly reduced in RTT brains (FDR = 1.07 × 10^−05^; Fig. 4).

### Compatibility with published transcriptional profiles

Two RNA-seq datasets of postmortem brain from females with RTT compared to controls have been published^13,14^ (Table 4). The first dataset examined pooled frontal and temporal cortex (FTTX) for each of three individuals with RTT compared to three CTL and is available from the Sequence Read Archive under accession number PRJNA302685^29^. The second larger dataset examined motor cortex (Motor) and cerebellum (Cblm) for nine females and six females with RTT, respectively, compared to eight CTL of each tissue, but the primary data were not accessible^14^. We downloaded the FASTQ files for the available dataset, aligned reads using salmon^30^ (see Code Availability 6), summarized the aligned reads into gene counts using tximport v1.12.1^31^ (see Code Availability 7), and retained genes with ≥10 counts in ≥3 samples. Count data were converted to logCPM to adjust for the total counts per sample using limma v3.40.2^32^ (see Code Availability 8), then observation-level and sample-level weights were estimated using voom^32,33^. We also reanalyzed our data using this workflow. Analysis of variance models were fit separately for each of three brain regions (CCTX, FCTX, FTTX), then combined in a random effects meta-analysis using GeneMeta v1.56.0^34^ (see Code Availability 9). Our meta-analysis identified 1,455 genes that were significantly differentially expressed (FDR < 0.05) between brain samples from control individuals and those with RTT.

**Table 4:** Published RTT Brain RNA-seq datasets.

Dataset	Brain Region	# Cases	# Controls	SRA	PMID
Lin	FTTX	2	3	PRJNA302685	27267200
	TCTX	1	–		
Gogliotti	Motor	9	8	–	29523700
	CBLM	6	8		

Abbreviations: CBLM, cerebellum; FTTX, pooled frontal cortex and temporal cortex from the same individual; Motor, motor cortex; TCTX, temporal cortex; SRA, sequence read archive; PMID, PubMed unique identifier for published reference article.

**Fig. 5:**
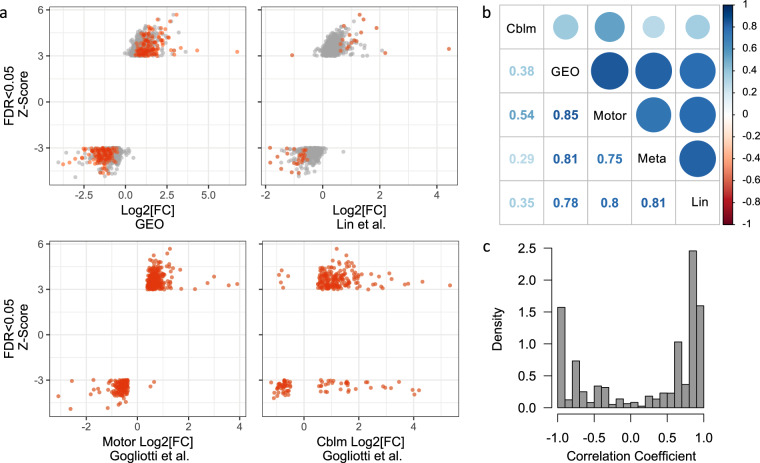
Replication of differential gene expression between RTT and CTL brain. (**a**) Meta-analysis Z-Score compared to log2 fold change (FC) between RTT and CTL from our initial analysis (GEO), Lin *et al*. combined frontal and temporal cortex (from Table S5)^13^, Gogliotti *et al*. motor cortex (from Table S2)^14^, and Gogliotti *et al*. cerebellum (from Table S3)^14^. Genes with significant differential expression (False Discovery Rate [FDR] < 0.05) in the dataset represented on the X-axis are in red. (**b**) Spearman’s correlation between meta-analysis Z-score and logFC for each of the other datasets. Color intensity and circle size are proportional to the correlation coefficients with values displayed below the diagonal. (**c**) Density of gene-wise correlation coefficients among datasets in (**b**).

To verify these results, we compared the results from our meta-analysis with differential gene expression results from previous RTT RNA-seq analyses^13,14^ (Fig. 5). We compared the Z-score for each of the significantly differentially expressed genes from our meta-analysis with the log2 fold change from our previous analysis (GEO DESeq2) and from each of the three published RNA-seq datasets (Lin *et al*., Gogliotti *et al*. Motor, and Gogliotti *et al*. Cblm; Fig. 5a). We found strong concordance among RTT transcriptional profiles from regions within the cerebral cortex, while RTT transcriptional profiles from the cerebellum were least correlated with the regions from the cerebral cortex (Fig. 5b). We aggregated the gene-wise correlation coefficients among datasets and found an overall positive correlation for 63% of the comparisons among datasets, indicating an overall agreement among the differential gene expression per dataset (Fig. 5c). Not only do our data represent an independent technical and biological replication of molecular alterations in RTT brain, but our meta-analysis demonstrates the power of combining datasets to maximize detectable results among several smaller studies.

## Supplementary information

Supplementary information

## Data Availability

We used the following software and versions to process our dataset as described in the text: 1. STAR v2.5.3a was used for mapping reads to the *Human* reference genome NCBI build 37/hg19: https://github.com/alexdobin/STAR 2. featureCounts v1.6 was used to summarize gene counts: http://bioinf.wehi.edu.au/featureCounts/ 3. Picard v2.15.0 was used to measure 5′ to 3′ bias: http://broadinstitute.github.io/picard 4. DESeq2 v1.20.0 was used for differential expression analysis: https://bioconductor.org/packages/release/bioc/html/DESeq2.html. 5. IGV v2.8.2 was used to visualize *MECP2* coding regions for sequence variation: http://software.broadinstitute.org/software/igv/ 6. Salmon was used to align reads to the *Human* GRCh38 reference transcriptome and estimate counts for each transcript: https://combine-lab.github.io/salmon/ 7. tximport v1.12.1 was used to summarize gene counts: https://bioconductor.org/packages/release/bioc/html/tximport.html 8. limma v3.40.2 was used to convert count data to log counts per million (logCPM) and to estimate weights: https://bioconductor.org/packages/release/bioc/html/limma.html 9. GeneMeta v1.56.0 was used to perform a random effects meta-analysis: https://www.bioconductor.org/packages/release/bioc/html/GeneMeta.html
