# Supplementary material for: Transcriptome data of temporal and cingulate cortex in the Rett syndrome brain
Source: Sci Data. 2020 Jun 19;7:192. doi: 10.1038/s41597-020-0527-2 (PMC7305197; doi:10.1038/s41597-020-0527-2)
Supplement: Supplementary file 1 — Supplementary information [file 41597_2020_527_MOESM1_ESM.docx]

**Supplementary Information**

**TABLE OF CONTENTS**

**Supplemental Figure 1 Page 2**

**Supplemental Figure 2 Page 3**

**Supplemental Figure 3 Page 4**

**Supplemental Figure 1.** Chromatograms showing Sanger sequencing confirmation for the *MECP2* c.473C>T (p.Thr158Met) intragenic variant reported in 7773 (CCTX, top; TCTX, bottom). DNA and protein sequences are shown. Forward primer used to sequence CCTX is shown (F – AAAGGTAGGCGACACATCCC, R – CAGTTCCTGGAGCTTTGGGA). Chromatograms were aligned to *MECP2* (ENSG00000169057), as implemented in Benchling (https://benchling.com).


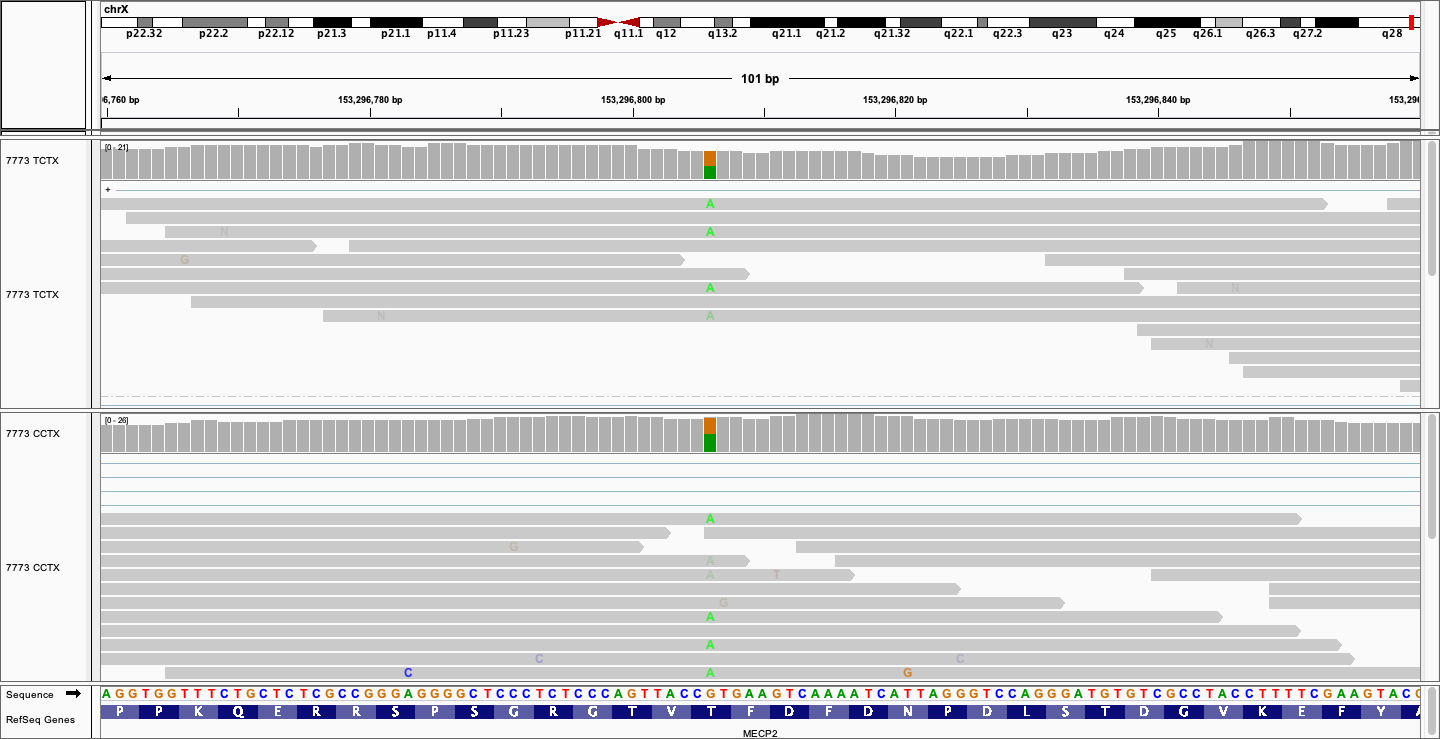


**Supplemental Figure 2.** *MECP2* c.473C>T (p.Thr158Met) intragenic variant in RNA-seq data from CCTX and TCTX for brain 7773 visualized through the integrative genomics viewer (hg19).


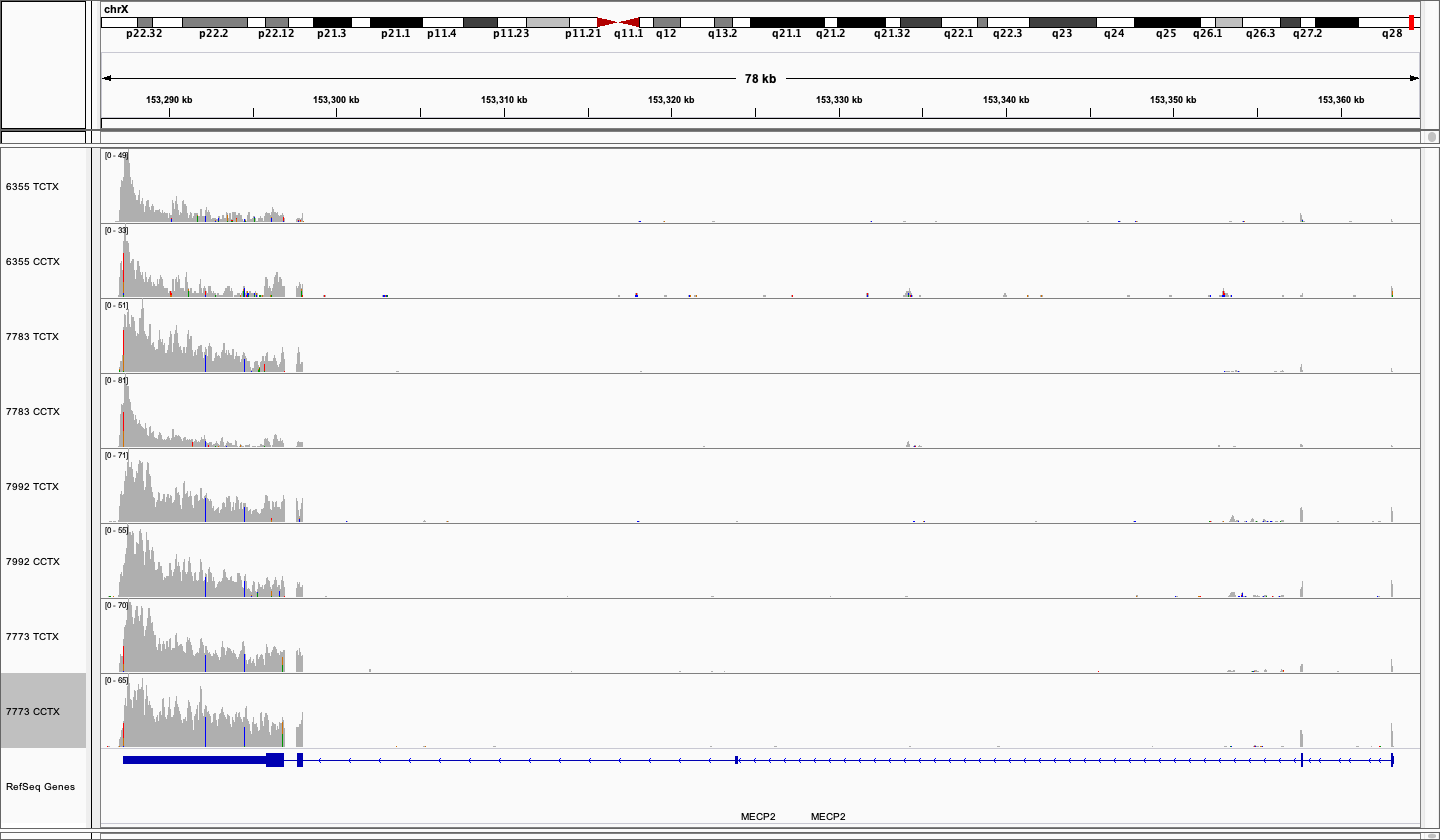


**Supplemental Figure 3.** *MECP2* RNA-seq coverage plots for each RTT brain sample visualized through the integrative genomics viewer (hg19).
